# STAT6 gain-of-function disease: p.D519N is a new disease-causing variant that responds well to dupilumab treatment

**DOI:** 10.1016/j.jacig.2025.100494

**Published:** 2025-05-09

**Authors:** Simran Samra, Eleanor Cook, Jessica Wilson, Mehul Sharma, Liam Golding, David T. Yao, Rebecca A. Marsh, Stuart E. Turvey

**Affiliations:** a Department of Pediatrics, British Columbia Children’s Hospital, The University of British Columbia, Vancouver, British Columbia, Canada; b Experimental Medicine Program, Department of Medicine, The University of British Columbia, Vancouver, British Columbia, Canada; c Division of Bone Marrow Transplant and Immune Deficiency, Cincinnati Children's Hospital Medical Center, Cincinnati, Ohio; d Department of Pediatrics, University of Cincinnati College of Medicine, Cincinnati, Ohio; e Kids Cancer Centre, Sydney Children’s Hospital, Sydney, Australia; f Pharming Healthcare, Inc, Warren, NJ

**Keywords:** STAT6 gain-of-function disease, inborn errors of immunity, primary atopic disorder, monogenic allergic disorder

## Abstract

This case report presents a new genetic variant that causes STAT6 gain-of-function disease in 2 patients, expands the clinical phenotype of STAT6 gain-of-function disease, and describes its authors’ success in using therapeutics to manage this rare disease.

Signal transducer and activator of transcription 6 (STAT6) is a transcription factor that drives allergic inflammation.^1^ Activation of STAT6 signaling by IL-4 or IL-13 cytokines leads to the differentiation of T_H_2 cells, enhances the survival and proliferation of B cells, and mediates class switching of immunoglobulins to IgE.^2^ STAT6 gain-of-function (GOF) disease is a recently discovered severe monogenic allergic disorder and thus an emerging area of research.^2^, ^3^, ^4^, ^5^, ^6^, ^7^ Here, we report a new disease causing STAT6 GOF genetic variant.

## Patient presentation

**Fig 1:**
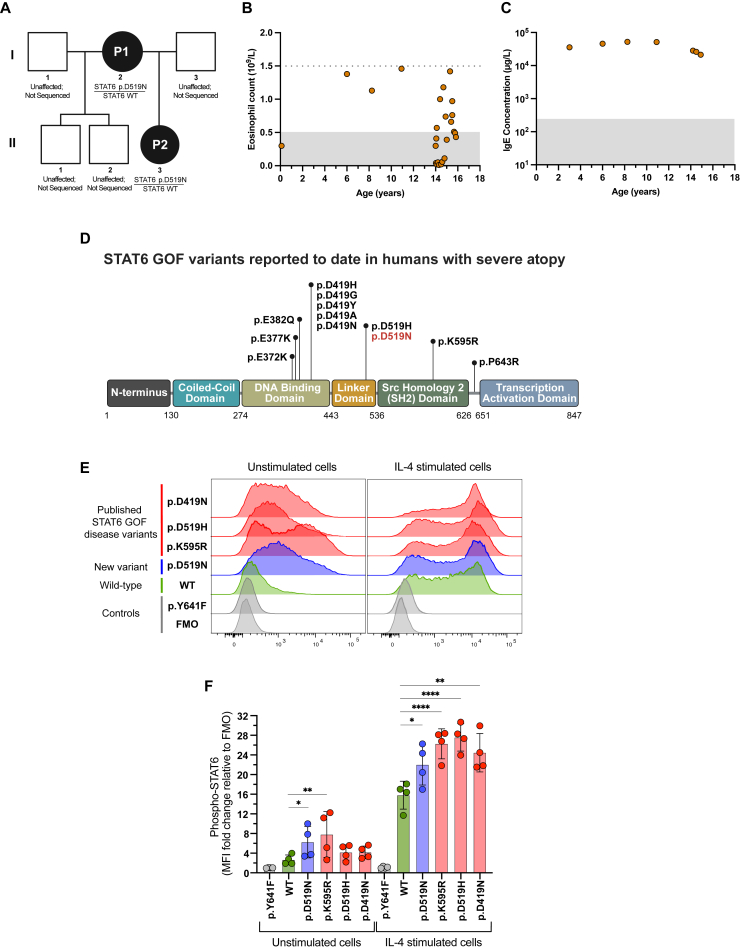
**A,** Family pedigree. **B** and **C,** The patient’s eosinophil (**B**) and IgE (**C**) counts in her whole blood. Shaded area represents normal counts, and horizontal broken line denotes the threshold for diagnosing hypereosinophilic syndrome. **D,** STAT6 GOF variants. **E,** Histograms showing STAT6 phosphorylation. **F,** Quantification of histograms using mean fluorescence intensity (MFI) (n = 4). Statistical comparisons based on 1-way ANOVA and the Dunnett multiple comparisons test. Stars denote *P* values as follows: ∗*P* < .05; ∗∗*P* < .01; and ∗∗∗∗*P* < .0001. *FMO*, Fluorescence minus 1; *WT*, wild type.

We report the case of a 15-year-old Black female with a history of mild childhood eczema, recurrent lower respiratory tract infections, and multiple food allergies (nuts, dairy, wheat, seafood, eggs, and some fruit and vegetables). The patient had markedly elevated absolute eosinophil counts and serum IgE levels (Fig 1, *A* and *B*). The results of her pulmonary function tests were abnormal and consistent with a moderate-severe restrictive defect. Her height and weight were within the normal range for her age (based on US Centers for Disease Control and Prevention data [normal stature for age and weight for girls aged 2-20 years]). Her lymphocycte subpopulations and vaccine titers were normal.

The patient had all the hallmarks of STAT6 GOF disease, early-onset severe atopic dermatitis, food allergies, and elevated absolute eosinophil counts and serum IgE levels. She also had additional symptoms observed in individuals with STAT6 GOF variants, including recurrent respiratory tract infections and anaphylaxis, but she had no history of gastrointestinal diseases such as eosinophilic esophagitis, asthma, or failure to thrive. She also did not present with lymphadenopathy, osteoporosis, joint hyperextensibility, or cerebral aneurysms (confirmed by a CT angiogram), which are less common features of STAT6 GOF disease.

The patient’s family history is notable. Her mother had a history of severe food allergies and recurrent respiratory infections, which are common symptoms of STAT6 GOF disease. Interestingly, the mother’s symptoms became milder with age. There is speculation that STAT6 GOF disease may improve with age; however, additional affected individuals need to be identified and monitored longitudinally.

The patient had 2 episodes of anaphylaxis as a young child. The frequency and severity of these anaphylactic reactions increased when she was around 14 years of age, prompting reengagement with specialty teams. She was initially prescribed omalizumab (300 mg every 4 weeks), which allowed for weaning off high-dose steroids (prednisone, 2 mg/kg per day). The patient has been taking famotidine (20 mg per day) and fexofenadine (180 mg twice per day) long term.

## Diagnosis and treatment

The patient and her mother provided consent for sequencing, data analysis, and publication of the findings. The patient was suspected of having an inborn error of immunity (IEI), a monogenic disorder in which parts of the human immune system are missing, dysfunctional, or poorly regulated. An IEI panel analyzing 429 IEI genes was used for next-generation sequencing of the patient’s genomic DNA. The results were noninformative, with no likely disease-causing changes found in the 429 IEI genes analyzed. Because of similarities between the patient’s clinical phenotype and STAT6 GOF disease (a recently identified IEI that was not a part of the IEI panel at the time of testing), the care team requested specific sequencing of the *STAT6* gene, including analysis for deletions and duplications. A germline heterozygous variant in the *STAT6* gene (NM_001178079.2) at position c.1555G>A (p.D519N) was found in the patient and later found to be inherited from her mother (Fig 1, *A*). STAT6 GOF disease has an autosomal dominant inheritance pattern, consistent with the inheritance pattern of the p.D519N variant passed from mother to child and disease development in both individuals.

The patient began taking dupilumab (300 mg every 2 weeks), which is a known effective treatment for patients with GOF *STAT6* variants.^2^^,^^8^ She has been taking dupilumab for almost a year. She experienced an initial improvement in symptoms with a year with no episodes of anaphylaxis in response to food (but 1 episode of anaphylaxis related to heavy cat hair exposure). In comparison, before she began taking dupilumab, the patient had multiple anaphylaxis episodes per year. Her infectious phenotype has also improved. Since starting dupilumab treatment, she has had only 1 mild illness (not requiring hospital presentation or inpatient management); in the year before she began taking dupilumab she had multiple respiratory illnesses, including 3 requiring admission to the intensive care unit (1 of which required noninvasive ventilation). Despite dupilumab treatment, the patient’s pulmonary function test results still show restrictive defects. More recently, she has experienced recurrent lip angioedema and an episode of anaphylaxis. As a result, she has begun treatment with the Janus kinase inhibitor ruxolitinib (5 mg twice per day).

## Functional analysis of the p.D519N variant

The p.D519N variant was suspected as the cause of the patient’s symptoms because a somatic version of the variant is documented in the Catalog of Somatic Mutations in Cancer (COSMIC). Of the 11 germline disease-causing STAT6 GOF variants reported in the literature, 9 were also previously documented in COSMIC. This trend is also observed in other IEIs caused by STAT proteins.^2^ Further evidence implicating the p.D519N variant as a disease-causing entity include another variant at the same amino acid position that is known to cause STAT6 GOF disease (p.D519H) (Fig 1, *D*).^3^ The p.D519N STAT6 variant was initially classified according to the American College of Medical Genetics and Genomics and Association for Molecular Pathology guidelines as a variant of uncertain significance.^9^ To confirm that the patient’s variant leads to a GOF in STAT6 activity in HEK293 cells, we performed functional experiments in which an established flow cytometry assay was used to measure STAT6 activity.^3^

HEK293 cells were transfected with the patient’s variant (p.D519N) by using 3 known STAT6 GOF disease variants (ie, p.D419N, p.D519H, and p.K595R),^3^ wild-type STAT6, or a biochemically inactive STAT6 variant (p.Y641F).^10^ The patient’s p.D519N variant, as well as the known STAT6 GOF disease variants, had markedly elevated STAT6 phosphorylation compared with wild-type STAT6 following IL-4 stimulation (Fig 1, *E* and *F*). According to the American College of Medical Genetics and Genomics and Association for Molecular Pathology guidelines,^9^ the STAT6 p.D519N variant can be classified as pathogenic, with supporting evidence for pathogenicity criteria PS3, PM2, PM5, PP1, PP3, and PP4.

Previous studies have established that elevated level of STAT6 phosphorylation is a GOF marker. STAT6 variants result in elevated phosphorylation level, increased transcriptional activity, and enhanced nuclear localization of STAT6 relative to wild-type STAT6.^2^, ^3^, ^4^, ^5^, ^6^, ^7^ Downstream, these variants upregulated *STAT6* target genes that cause proallergic immune responses as well as increased levels of T_H_2 cytokines and STAT6-regulated chemokines, which is consistent with a GOF in STAT6 function.^2^, ^3^, ^4^, ^5^, ^6^, ^7^

## Recommendations for clinicians

We anticipate that new disease-causing genetic variants in *STAT6* will continue to be discovered. Clinical and research-based assays to quantify STAT6 phosphorylation may not be widely available for clinicians who have a patient with a novel *STAT6* variant. In these situations, segregation of the variant through the family and checking of whether the variant is documented in COSMIC may be informative in making a presumptive diagnosis of STAT6 GOF disease.

## Disclosure statement

Supported by grants from the Canadian Institutes of Health Research (grant PJT-178054 [to S.E.T.]) and 10.13039/501100000233Genome British Columbia (grant SIP007 [to S.E.T.]). S. E. Turvey holds a Tier 1 Canada Research Chair in Pediatric Precision Health and is an Aubrey J. Tingle Professor of Pediatric Immunology. S. Samra is supported by a 10.13039/501100005247University of British Columbia 4-year doctoral fellowship.

Disclosure of potential conflict of interest: R. A. Marsh holds part-time employment at Pharming Healthcare, Inc, and does advisory board work for Amgen, SOBI, and Sumitomo Pharma. The rest of the authors declare that they have no relevant conflicts of interest.
